# Smart Nanocarriers as an Emerging Platform for Cancer Therapy: A Review

**DOI:** 10.3390/molecules27010146

**Published:** 2021-12-27

**Authors:** Madhuchandra Kenchegowda, Mohamed Rahamathulla, Umme Hani, Mohammed Y. Begum, Sagar Guruswamy, Riyaz Ali M. Osmani, Mysore P. Gowrav, Sultan Alshehri, Mohammed M. Ghoneim, Areej Alshlowi, Devegowda V. Gowda

**Affiliations:** 1 Department of Pharmaceutics, JSS College of Pharmacy, JSS Academy of Higher Education and Research, Mysore 570015, India; madhuchandra152@gmail.com (M.K.); sagarguruswamy223@gmail.com (S.G.); riyazosmani@gmail.com (R.A.M.O.); gowrav@jssuni.edu.in (M.P.G.); 2 Department of Pharmaceutics, College of Pharmacy, King Khalid University, Abha 61421, Saudi Arabia; shmohamed@kku.edu.sa (M.R.); ummehaniahmed@gmail.com (U.H.); ybajen@kku.edu.sa (M.Y.B.); 3 Department of Pharmaceutics, College of Pharmacy, King Saud University, Riyadh 11451, Saudi Arabia; salshehri1@ksu.edu.sa; 4 Department of Pharmacy Practice, College of Pharmacy, AlMaarefa University, Ad Diriyah 13713, Saudi Arabia; mghoneim@mcst.edu.sa (M.M.G.); ashlowi@mcst.edu.sa (A.A.)

**Keywords:** cancer, smart nanocarriers, drug targeting, nanoparticles, stimulus for drug release

## Abstract

Cancer is a group of disorders characterized by uncontrolled cell growth that affects around 11 million people each year globally. Nanocarrier-based systems are extensively used in cancer imaging, diagnostics as well as therapeutics; owing to their promising features and potential to augment therapeutic efficacy. The focal point of research remains to develop new-fangled smart nanocarriers that can selectively respond to cancer-specific conditions and deliver medications to target cells efficiently. Nanocarriers deliver loaded therapeutic cargos to the tumour site either in a passive or active mode, with the least drug elimination from the drug delivery systems. This review chiefly focuses on current advances allied to smart nanocarriers such as dendrimers, liposomes, mesoporous silica nanoparticles, quantum dots, micelles, superparamagnetic iron-oxide nanoparticles, gold nanoparticles and carbon nanotubes, to list a few. Exhaustive discussion on crucial topics like drug targeting, surface decorated smart-nanocarriers and stimuli-responsive cancer nanotherapeutics responding to temperature, enzyme, pH and redox stimuli have been covered.

## 1. Introduction 

Cancer is defined as uncontrolled cell growth and the lack of cell mortality, resulting in an abnormal cell mass, i.e., tumour, apart from haematological malignancy, where tumour cells multiply and proliferate throughout the lymph, blood and bone marrow systems [1]. Chemotherapy is the use of chemicals to kill or inhibit tumour progression, because tumour cells develop considerably faster than normal cells, and chemotherapy medications target those rapidly developing cells. However, some of the normal cells are also growing rapidly, so chemotherapy drugs target those rapidly multiplying normal cells [2,3]. The destruction or alteration of proto-oncogenes, which encode proteins involved in cell growth and division and tumour suppressor genes, which encode proteins that give inhibitory signals to cell development and trigger cell death, are the most common causes of cancer. Mutations in tumour susceptibility genes, which code for proteins involved in DNA damage regulation, are required for tumour formation and are encouraged by mutations in oncogenes and tumour suppressor genes. The mutations which cause tumours are clonally chosen to favour abnormal and unregulated cell growth, the lack of abnormal cell growth inhibition, the minimization of the immune system, the obliteration of cell mortality and transmission and the build-up of genetic information defects [1,4].

Although radiotherapy and surgery are the most appropriate and beneficial therapies for non-metastatic and local malignancies, they are ineffective when the tumour cells are spread to other parts of the body. Cancer medications (such as biological, chemotherapy and hormonal treatments) may reach each organ in the body through the circulation, they are the present treatment of options for metastatic cancers [1,5]. Conventional medicines have minimal aqueous solubility, bioavailability and therapeutic benefits. In greater doses, this substance is required to cause toxicity. The advancement of nanotechnology has a significant impact on cancer treatment [6]. 

To recognize tumour regions nanocarriers use physiochemical differences between tumour and normal cells. There are two methods for determining the location of tumour cells. The Enhanced Permeability (EPR) effect is used in passive targeting to determine the tumour location indirectly. Cancer cells are killed by employing an overexpressed cell surface receptor as a guided missile in active targeting. The next stage is to deliver medications in a specific place and at a specific concentration. Based on the nature and intelligence of the nanocarriers, drugs can be delivered by internal or external stimuli [3,7]. Smart-nanocarriers are colloidal nano-scale particles capable of delivering anticancer drugs, such as medicine, that contain low molecular weight components such as genetics or enzymes [1]. Nanocarriers (10–400 nm) were chosen as drug carriers because of their ability to carry large amounts of medication, provide prolonged flow times and preferentially target tumour location due to enhanced permeability and retention effect (EPR). The P-glycoprotein is a drug efflux transporter that is commonly expressed on the surfaces of tumour cells produces MDR (multidrug resistance), smart-nanocarriers are used to combat MDR [8,9]. This review overviews the current advances in smart nanocarriers such as dendrimers, liposomes, mesoporous silica nanoparticles, quantum dots, micelles, superparamagnetic iron-oxide nanoparticles, gold nanoparticles and carbon nanotubes. This review discusses various topics like drug targeting, smart-nanocarriers and targeting moieties that respond to several stimuli including temperature, enzyme, pH and redox stimulus.

## 2. Drug Targeting

Smart-nanocarriers which are used for tumour targeting result in improved drug release, increased intracellular and internalization delivery, pharmacodynamic and pharmacokinetic profiles, controlled and higher specificity and most importantly lowers toxic effects [8]. Some of the common features of tumours are leaky blood vessels and poor lymphatic drainage. Two important types of drug targeting include active targeting and passive targeting.

### 2.1. Passive Targeting

**Figure 1:**
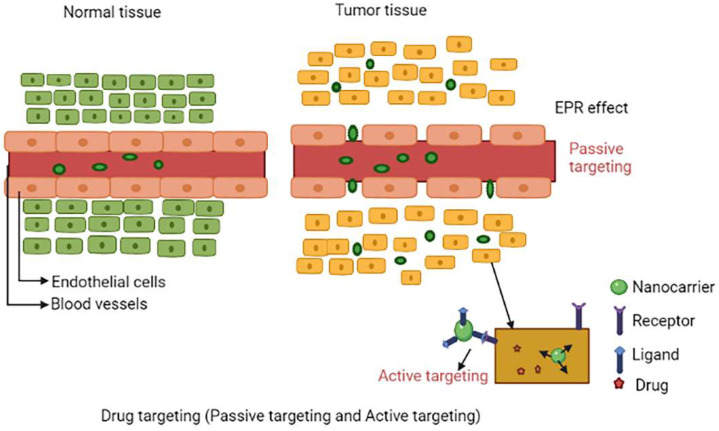
Schematic representation of drug targeting via passive targeting (EPR effect) mode and active targeting mode.

Passive targeting to tumour cells can be done by EPR effect, which is exhibited by tumour cell. Due to the leaky endothelium of the tumour vasculature, the rate of drug-loaded nanocarriers accumulating in a tumour is substantially greater than the healthy tissue. This is referred to as enhanced permeability effect. A defect in the lymphatic system causes nanoparticle retention in the tumour. This is referred to as the enhanced retention effect. The EPR effect refers to both phenomena [10]. Passive targeting is mostly determined by carrier characteristics such as tumour leakiness and vascularity, as well as size and circulation time. When compared to healthy organs, passive targeting significantly improves in specificity by 20–30%. Furthermore, EPR-based passive targeting to tumours is influenced by nanocarrier features like charge, size and surface chemistry, as well as the limitations imposed by improbable cell targeting within malignant tumours [11,12]. The EPR effect will be excellent if smart-nanocarriers can avoid immune surveillance and circulate for a long time. At the tumour location, very relatively high concentrations of drug-loaded smart-nanocarriers can be achieved in 1–2 days, 10–50 times higher than in normal cells [13]. Figure 1 shows the schematic representation of drug targeting via passive targeting mode and active targeting mode.

Hansen et al. created copper-64-loaded liposomes (PEGylated) and used in imaging to evaluate their EPR effects. Despite the fact that EPR had a dominant effect in only a few tumours, the outcome of high liposome deposition in 11 dogs with various solid tumours could not be extrapolated to any tumour [14]. Interstitial fluid pressure (IFP) is one obstacle to the efficient deposition of drug-incorporated nanocarriers in cancer cells [15]. Successful nanocarrier advancements can overcome various biological obstacles, such as IFP (interfacial fluid pressure) and RES (reticuloendothelial tissues) [16].

### 2.2. Active Targeting

Tumour cells and surface-modified targeted nanoparticles are used in active targeting [12,17]. Certain on-surface cells, such as cell surface antigens and folic acid, have been shown to be increased and overexpressed by tumour cells. Active ligands are coupled with drug-induced nanocarriers, where these ligands will recognise their overexpressed target on the surface of tumour cells. Aptamers, transferrin, peptides, folate and antibodies are the most commonly studied ligands [3]. Immunoliposomes, or antibody conjugated liposomes, are another technique in the active targeted delivery of anti-tumour medicines. Immunoliposomes, like liposomes, encapsulate anti-tumour medicines, but due to the associated tumour-specific antibody, they provide high concentration cancer cell targeting. Doxorubicin-loaded anti-human epidermal growth factor receptor-2 immunoliposomes have been proven to have a higher therapeutic effects against numerous breast cancer, when compared to naked PEGylated liposomes [18].

## 3. Nanocarriers Used in Cancer Therapy

The nanoparticles (NPs) have recently received a lot of interest because of their drug carrier systems, bio-medicine potential as targeting systems, bio-imaging and controlled drug releases. Functional organic solutes are typically encapsulated into NPs to overcome their limited water solubility. The hydrophilic coatings on Nanoparticle surfaces can also be coupled with amphiphilic surfactants, allowing insoluble organic solutes to be readily supplied and distributed in an aqueous phase [19,20]. Nanocarriers protect medications from degradation, reduce their half-life in the bloodstream and renal clearance, increase the utility of cytotoxic medications, regulate the release kinetics of antitumour medications and increase the solubility of chemicals [1]. In terms of structure and intelligence, several fascinating smart-nanocarriers are described in detail below.

### 3.1. Liposomes

Liposomes are phospholipid-enclosed concentric bilayer vesicles with a hydrophilic centre [21]. Since they may entrap a wide range of medicines, both hydrophilic and lipophilic in nature, liposomes have been intensively researched as a preferred carrier for the delivery of therapeutic drugs in recent decades [22,23,24]. Liposomes have various advantages, including active group protection, cell-like membrane structure, minimal immunogenicity, biocompatibility, safety, efficacy and increased half-life [25]. Although, typical liposomes are also having drawbacks, including low entrapment and a higher likelihood for hydrophilic and amphiphilic medications to escape from liposomal vesicles, as well as accelerated blood clearance (ABC) through the reticuloendothelial system (RES). When liposomes are recognized as foreign to the body resulting in taken up by the mononuclear phagocyte system (MPS) and RES macrophages. The physicochemical characteristics of liposomes such as size, charge, hydrophilicity and hydrophobicity all influence their removal from the body’s systemic circulation. PEGylated liposomes solve the RES uptake problem. Then, targeted liposomes were created to allow for the selective delivery of drugs to the appropriate region. Peptide, transferrin, folate, mannose, antibody and asialoglycoprotein are some of the ligands used in liposome targeting [26,27]. Using stimuli-triggered drug delivery systems, components of the tumour microenvironment (such as hypoxia, slightly increased temperature and acidic pH) have recently been utilized to deliver payloads in tumour tissues [28,29,30,31,32]. Drug localization, bio-distribution and therapeutic efficacy can all be tracked using theragnostic systems, which incorporate both a diagnostic and a therapeutic moiety in a liposomal system [33].

The primary issue with using liposomal delivery systems are ABC and RES uptake. There are many efforts are made to prevent liposomes uptake by RES and to increase the systemic circulation duration through a size adjustment or liposome surface modification. Liposomes of the second generation are a sort of customized liposome containing oligosaccharides, glycoproteins, synthetic polymers and polysaccharides are added to the surface to enhance circulation time. To achieve extended blood circulation of liposomes, many approaches have been used such as PEG coating on the liposomal surface. These PEGylated-liposomes demonstrated improved blood circulation time, greater biodistribution, good stability and better antitumour effectiveness (Long circulatory liposome) [34,35].

**Figure 2:**
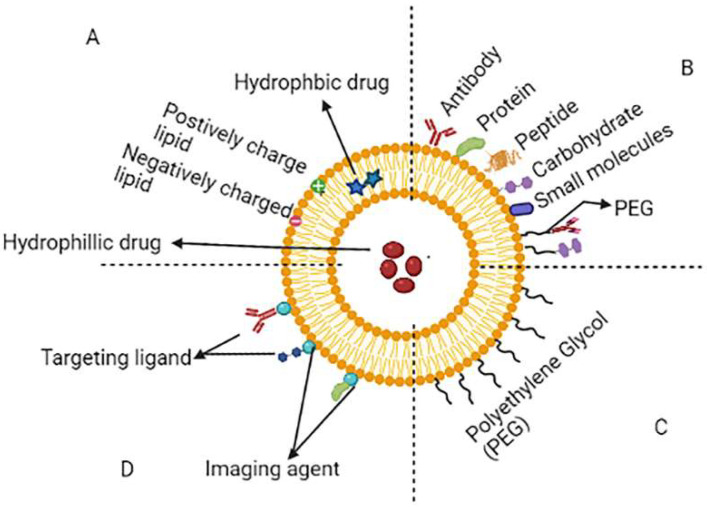
Schematic representation of different types of liposomes. (**A**) Conventional liposome, (**B**) ligand targeted liposome, (**C**) PEGylated liposome and (**D**) theranostic liposome.

The pH of the tumour microenvironment has been found to be different from the pH of healthy cells as a result, using a pH-sensitive liposomal formulation to increase medication accumulation at the tumour site (extracellular or intracellular) could be a potential strategy [36]. The pH-stimuli liposomes can stay constant at physiological-pH (pH 7.5), but once inside the tumour (PH 5.7) causes pH-triggered drug release due to lipid layer break down [37]. The hyaluronic acid targeted PH-stimuli liposomes were produced and shows improved effectiveness against CD44 receptor overexpressing cells and lower toxic effects towards healthy cells than free doxorubicin (stimuli sensitive liposome) [38]. The schematic representation of different types of liposomes are shown in Figure 2. The FDA approved liposomal formulation for cancer therapy as show in Table 1.

### 3.2. Dendrimers

**Figure 3:**
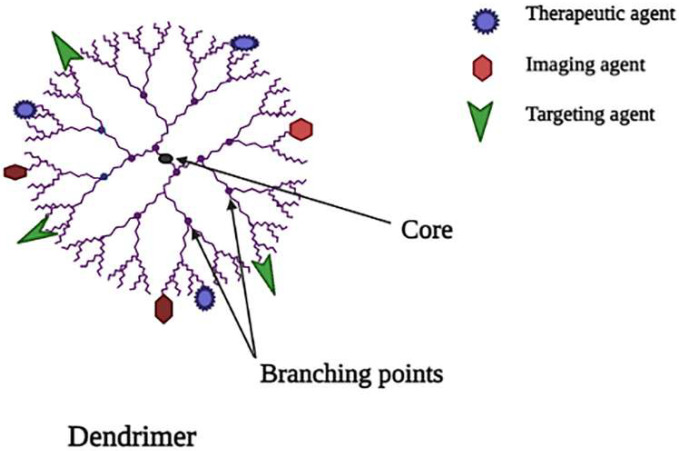
Schematic representation of dendrimer.

Dendrimers are also known as dendron, which is derived from a Greek word, which means “tree”, since it has a similar branching structure as a tree [42]. Dendrimers are made up of three main components [1]. A unit that repeats itself and is linked to the central core; these layers are called generations because they are radially homocentric [2,3]. A central core of pharmacokinetic profiles and biocompatibility are determined by a functional group at the dendrimer’s periphery [43]. Since the cationic dendrimers cause cell lysis, which damages the cell membrane due to interaction between the negatively charged cell membrane and the positively charged dendrimer surface, PEGylation and glycosylation enhance dendrimer biocompatibility [44,45]. Figure 3 shows the schematic representation of dendrimers.

As nanocarriers for cancer therapy, poly-amidoamine (PAMAM), poly-l-lactide, poly-lysine, peptide dendrimer, poly-propylene-imine, poly-caprolactone and poly-ethylene glycol are currently employed [46]. For cancer treatment, paclitaxel, doxorubicin (DOX), methotrexate and cisplatin are loaded into dendrimer nanosystems, as are iron oxide nanoparticles and gold nanoparticles incorporated in dendrimer for imaging and diagnostic [47]. By connecting specific molecules to the dendrimer, which has the potential to attack cancer cells, the efficiency of cancer therapy can be increased, resulting in a decrease in toxicity and aiding in the control of cancer therapy side effects. Typically, ligands (galactose, Dextran and folate) and antigens are used as targeting molecules [48]. These ligands are used to deal with the cationic toxicity of dendrimers, as well as to target tumour cells [49]. 

Investigators are currently investigating stimuli-responsive dendrimers, in which drug release happens when a specific stimulus is delivered by the external environment. Temperature, magnetic, light, pH and other sorts of stimuli are available, with dendrimer being responsive to pH-sensitive [50]. The dendrimers which are PH-sensitive used for cancer cell-specific delivery have hydrolysable connections that remain intact during circulation, but disintegrate quickly once inside the cancer cell, releasing medication for anticancer action [51].

Poly-amidoamine dendrimer loaded with doxorubicin conjugation for tumour therapies was reported by Lai et al.; at 4.5 pH, nanocarriers release drugs faster (47 percent in 24 h) than at 7.4 pH (8 percent in 24 h), although PAMAM-amide-DOX releases drugs slower than PAMAM-hyd-DOX at 4.5 pH. PAMAM-hyd-DOX dendrimer nanocarriers are more harmful to malignant cells than PAMAM-amide-DOX nanocarriers [52] as shown in Table 2.

### 3.3. Micelles

**Figure 4:**
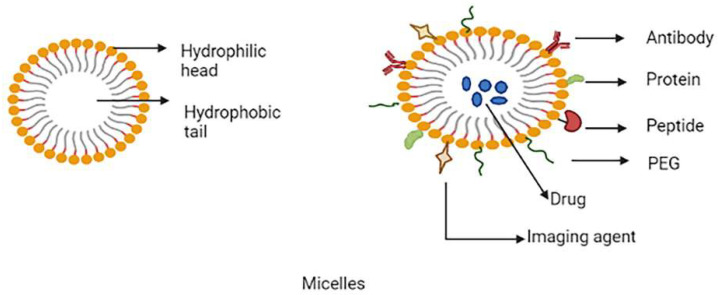
Schematic representation of multifunctional micelles.

Polymeric micelles have gained popularity in recent years and have become one of the most well-studied nanocarriers in cancer detection and treatment. These micelles are made up of spherically shaped, self-assembled amphiphilic block co-polymers with a hydrophilic corona and the hydrophobic core in an aqueous medium with a diameter ranging from 10–100 nm. Hydrophobic drugs can be accommodated in the core of the micelle [54,55]. In active targeting of tumour cells, several kinds of ligands, such as aptamers, peptides, antibodies, carbohydrates, folic acid, etc., are utilized to decorate the micelle surface. The stimuli-based micelle drug delivery systems are based on enzymes, ultrasound, temperature changes, PH gradient and oxidation [56]. To enhance intracellular uptake, a variety of functional groups can be attached to the micelle’s hydrophilic end. The active components of the pH-sensitive polymeric micelle are generally released at lower pH [57]. The co-delivery technique, which employs a multifunctional micelle, is critical for the synergistic benefits in tumour therapies. The temperature-stimuli micelle-based co-delivery system described by Seo et al. is capable of transporting genetics as well as anti-tumour medications [58]. Polyion complex (PIC) micelles are a type of micelle that is being researched primarily for the efficient delivery of genes and siRNAs [59]. The schematic representation of multifunctional micelles as shown in Figure 4.

Wan et al. conducted a study of designer polymeric micelles for targeting ovarian and breast cancers, which featured simultaneous loading of paclitaxel and cisplatin in amphiphilic copolymer-based micelles, which resulted in a considerable increase in loading efficiencies [60]. P-glycoprotein (P-gp) is an efflux transporter, The efflux of diffused intracellular anticancer medicines is mostly caused by overexpression of P-gp in tumour cells, resulting in low bioavailability of the drug. Razzaq, S et al. developed a mucopermeating papain functionalized thiolated redox micelle for site-specific administration of paclitaxel, that the developed formulations can inhibit P-gp efflux pump, improve oral bioavailability, higher penetration and enhanced efficacy compared to conventional paclitaxel formulation [61]. The different types of polymeric micelle for cancer therapy used in clinical trials are shown in Table 3. 

### 3.4. Carbon Nanotubes (CNTs)

**Figure 5:**
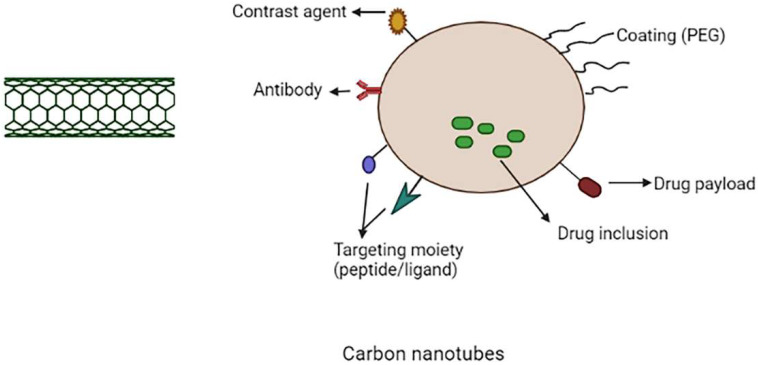
Schematic representation of multifunctional carbon nanotubes.

CNTs are carbon-based cylindrical molecules that can be employed as nanocarriers in cancer therapy. CNTs are produced from graphene sheets rolled into a seamless cylinder with a high aspect ratio, diameters as small as 1 nm and their lengths can reach up to several micrometres and they can be open-ended or capped [63]. The two types of carbon nanotubes are single-walled carbon nanotube (SWCNTs) and multi-walled carbon nanotubes (MWCNTS). Single-walled carbon nanotubes are single graphene cylinders, whereas multi-walled carbon nanotubes are a complex nesting of graphene cylinders. SWCNTs have a smaller diameter, are more flexible and can help with imaging. On the other hand, MWCNT’s have a large surface area and so the endohedral filling is more efficient [64,65,66]. Carbon nanotubes received more attention among other carbon-based nanocarriers and spherical nanoparticles due to their distinctive properties such as intracellular bioavailability, high cargo loading and ultra-high aspect ratio [67,68]. The schematic representation of multifunctional CNTs are shown in Figure 5.

CNTs have been utilized in a variety of applications, including anticancer drug delivery and gene therapy. Non-spherical nanocarriers like carbon nanotubes can stay in lymph nodes for longer than spherical nanocarriers like liposomes [63]. According to Yang et al., CNTs could be utilised to target lymph node tumours. In this study, FA-functionalized MWCNTs were used to entrap magnetic nanoparticles incorporated with cisplatin. The nanotubes were dragged to the lymph nodes using an external magnet and the drug release was achieved for several days in the tumour cells [69,70]. To make CNTs smart, they should be functionalized chemically or physically [71]. PEGylation is a critical step in increasing solubility, avoiding RES and reducing toxicity [72]. 

Another area of research that is now being investigated is the use of functionalized carbon nanotubes as a nanocarrier for gene therapy. Biomolecules such as miRNA, siRNA, dsDNA and others, in comparison to small molecule drugs, cannot enter cellular membranes and are quickly breakdown by nucleases [68,73]. On the surfaces of carbon nanotubes both RNA and DNA can easily accommodate, improve the therapeutic efficacy of aptamers, micro-RNA (miRNAs) and small interference RNA (siRNAs), oligonucleotides and double-stranded DNA (dsDNA) and because of their extraordinary flexibility and structure, carbon nanotubes can also carry large amounts of genetic materials to targeted areas [74,75]. The different types of CNTs used for cancer therapy are shown in Table 4. In the treatment of cancer, immunotherapy may be an alternative to gene therapy. SWNTs were coated with tumour-specific fluorescent probe, radiometal ion chelates and monoclonal antibodies. A variety of approaches have been shown to be capable of targeting the tumour (lymphoma) [76].

### 3.5. Gold Nanoparticles (AuNPs)

**Figure 6:**
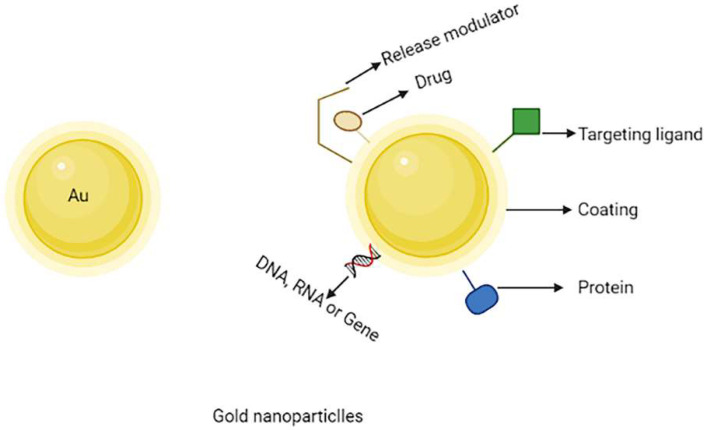
Schematic representation of multifunctional gold nanoparticles.

AuNPs have received current scientific interest among numerous nanocarriers developed for use in nanomedicines due to their unique uses in cancer therapy such as drug delivery, tumour sensing and photothermal agents [78]. For a variety of reasons, the use of AuNPs in cancer treatment and diagnosis is gaining a lot of interest. Furthermore, their inactivity toward biological systems has made them superior to conventional metal-based drug delivery technologies [79]. The inorganic nanoparticles have non-sensitive physical-chemical properties and are meant to convert irradiation energy into harmful radicals for photodynamic or photothermal therapy for solid malignancies. Due to their unique features, inorganic nanoparticles serve an important role in a variety of domains, including drug processing, bioimaging and sensing. Inorganic nanocarriers such as gold nanoparticles perform an essential pharmacological role. When AuNPs are adjusted to a proper shape and size, they are likewise non-toxic and have low phototoxicity [80,81]. The schematic representation of multifunctional gold nanoparticles are shown in Figure 6.

The optical properties, tuneability and surface plasmon resonance of gold nanoparticles drew researchers’ attention nowadays. AuNPs can be modified easily by changing the appearance and applying a negative charge on the gold nanoparticles surface. This means that by combining various molecules such as ligands, medicine and genes can be easily functionalized. Furthermore, the non-toxicity and biocompatibility of gold nanocarriers make an excellent choice for utilizing as a drug carrier, for example, when methotrexate coupled with gold nanoparticles, which has been used to treat cancer, has shown to be more cytotoxic to a variety of tumour cell lines compared to free methotrexate. MTX was observed to rise at a faster rate and a higher concentration in tumour cells when conjugated with gold nanoparticles. When coupled with gold nanoparticles via an acid-labile connection, doxorubicin is a marker of enhanced toxicity to the MCF-7/ADR breast cancer cell line, which is multidrug-resistant [82,83,84]. 

PEGylated gold nanoparticles can overcome the problem of RES uptake. Under physiological conditions, PEGylated-gold nanoparticles have better stability and solubility. The surface of gold nanoparticles could be modified to allow for targeted medication delivery via various ligands. For example, gold nanoparticles conjugated to fluorescent heparin might be utilised for cancer diagnostics and transferrin could be conjugated on the surface of gold nanocarriers for targeting [85]. To improve the effect of limited photodynamic therapy, Xin et al. created phthalocyanine chloride tetra sulphonic acid (AlPcS4) delivery systems using AuNPs. As AuNPs are not only easily accessible to AlPcS4, but also exhibit accelerated single oxygen production and directly cause cell death with photothermal effects, AlPcS4 has a significant anti-tumour action [86]. 

Apart from the synthetic approach of synthesising NPs, recently the herbal or biogenic approach has got much attention by the researchers and is been widely explored. In one such attempt, Xing et al. have studied innovative chemotherapeutic AuNPs to treat bladder cancer in a recent study and the AuNPs were prepared using *Citrus aurantifulia* seed extract. The outcomes of the clinical trial established that the AuNPs can be used as antioxidant, anticholinergics, anti-diabetic and anti-bladder cancer supplements in humans [87]. The biogenic nanoparticles are devoid of chemical neurotoxicity being of natural origin and hence are considered as the safest mode of augmenting cancer therapy with a reduced degree of toxicity. The applications of AuNPs in drug delivery for cancer therapy are shown in Table 5.

### 3.6. Mesoporous Silica Nanoparticles (MSNs)

**Figure 7:**
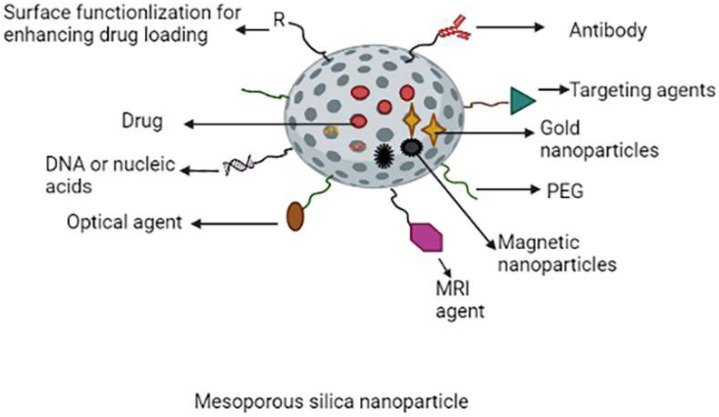
Schematic representation of multifunctional mesoporous silica nanoparticles.

Due to their extraordinary potential as nanocarriers for cancer therapy and imaging, mesoporous silica nanoparticles have received the attention of researchers [89,90,91,92,93,94]. MSNs have been studied and found to be promising carriers for biomedical imaging and drug delivery due to their good biocompatibility, high pore volume, uniform pore size distribution, large surface area and further chemical modification on the surface of MSNs to modulate the nanoparticle surface characteristics. Furthermore, pharmaceuticals can be placed onto the mesoporous, resulting in prolonged drug release [94,95]. Mesoporous sizes range from 2 to 50 nm. MCM-41 nanoparticles were the most extensively described MSNs for cancer therapy. This class of MSN is hexagonally structured homogeneous mesoporous that facilitates drugs to be loaded into micro-channels while also inhibiting the pre-release of loaded drugs [2,96]. On surfaces of the amine groups of MSNs, polyethylene glycol was conjugated to create long-circulation MSNs [97]. The Schematic representation of multifunctional mesoporous silica nanoparticles are shown in Figure 7.

For tumour cell targeting, several targeting ligands such as transferrin, mannose and folic acid (FA) have been coupled on surfaces of the MSNs. For example, the folate receptor (FR), which is typically overexpressed in many human tumour cells, has been widely employed in targeting the tumour cells and nanomaterial treatment. Researchers used an amide linkage to conjugate folate with polyethyleneimine and then this co-polymer coated with silica particles. When compared to non-targeted nanoparticles, FA-modified silica nanoparticles showed increased cytotoxicity in both human cervical and breast cancer cells and tumour absorption [98,99,100]. MSNs are employed in nucleic acid-guided treatments and nucleic acid delivery because of their relatively large surface area, superior biocompatibility for functionalization and variable pore size used to encapsulate various cargos [101,102,103,104].

MSNs have recently been developed as nanocarriers for photodynamic therapy (PDT), photothermal therapy (PTT), or both. PTT and PDT, two important types of phototherapies, sparked a lot of interest in various cancer treatments [105]. The applications of MSNs are shown in Table 6. 

### 3.7. Superparamagnetic Iron Oxide Nanoparticlesd (SPIONs)

**Figure 8:**
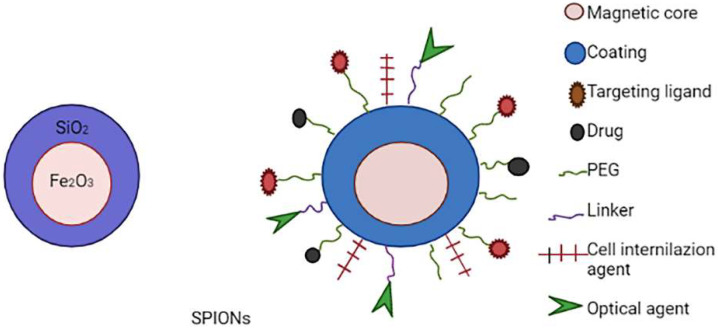
Schematic representation of multifunctional SPIONs.

SPIONs have become one of the most intensively investigated targeted nanomaterials because of their exceptional super-paramagnetic capabilities, which allow them to aggregate in a specific tissue under an external magnetic field [107]. When exposed to an alternating magnetic field (AMF), SPIONs have excellent magnetic resonance imaging (MRI), photothermal and magnetic heating capabilities, as well as strong biocompatibility. All of these characteristics make them promising candidates for use as a drug delivery system, a contrast agent in MRI and a thermotherapy agent [108,109]. SPIONs, on the other hand, have limited use since they agglomerate and are not stable in aqueous solutions. The constraint could be overcome by covering the SPION surface with various materials to change its surface properties [110]. The optimal size of nanoparticles in drug delivery systems based on SPIONs for in vivo applications should be between 10 and 200 nm, which allows them to avoid extravasation and renal clearance (<10 nm) and escape the attack of reticuloendothelial system macrophages (>200 nm) [111]. The schematic representation of multifunctional SPIONs are shown in Figure 8.

Polymers, liposomes, inorganic nanoparticles and viral vectors, including adenoviruses, have typically been conjugated with SPIONs. Surface modification of SPIONs has recently resulted in remarkable development in the field of magnetic nanoparticle-based nonviral medication delivery systems [112,113]. Such systems can deposit in the tumour region via superparamagnetic SPION capabilities in the presence of an external magnetic field (active) or by the enhanced permeability and retention effect (passive) [114]. Conjugation of SPION-based drug delivery systems with targeting moieties such as antibodies, hyaluronic acid, transferrin, peptides, folate and targeting aptamers (e.g., Arg-Gly Asp (RGD)) provides an alternate technique for improving targeting performance. Certain integrins/receptors that are overexpressed on the tumour cell surface can be detected by these targeting moieties, resulting in dose reduction and off-target effects [115]. The SPIONs used or under clinical trials for cancer therapy are shown in Table 7. 

### 3.8. Quantum Dots (QDs)

QDs are inorganic nanoparticles that have electrical, optical and fluorescent capabilities by nature. With the proper modifications, QDs are water-soluble and can be produced in sizes like 2–4 nm [117]. This nanocarrier could be utilised to visualise the tumour while the drug is being delivered to the desired location. A core, a shell and a capping substance are the three parts of commercially available QDs. A semiconductor material, such as CdSe, is used for the core. The semiconductor core is surrounded by a shell made of another semiconductor, such as ZnS. The double-layer QDs made of various substances are encapsulated by a cap [118]. In physiological systems the performance of quantum dots can be improved by functionalizing with biocompatible polymeric materials (PEG) or biological targeting molecules (antibodies) on the surfaces of quantum dots [119,120,121,122]. 

**Figure 9:**
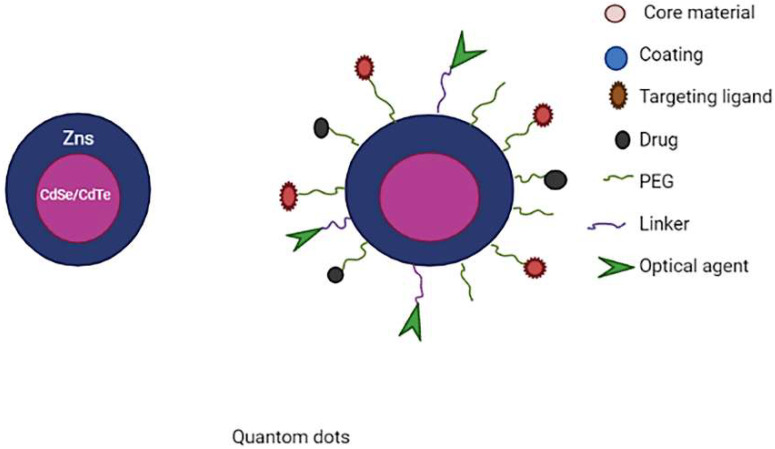
Schematic representation of multifunctional quantum dots.

Graphene quantum dots (GQD), carbon quantum dots (CQD) and cadmium-based QDs are the most often used QDs. Cadmium derivatives, like cadmium sulphide (CdS) and cadmium selenide (CdSe), are the most often utilised for core materials. These systems have been thoroughly investigated in terms of toxicity, size, photoluminescence, morphology, biocompatibility and stability [123]. Substances such as telluride and selenium give the system semiconductor and optical characteristics, making QDs semiconducting [124]. The usage of graphene-based QDs in targeting tumour cells and imaging has increased due to overcoming the cadmium-related toxicity problems. G-QDs can be further modified to increase their targeting towards a certain tumour cell type, making them more appealing for cancer subtype mapping and site-specific imaging [125]. Carbon QDs are the new types of nanostructures with the ability to replace conventional dots due to superior features such as photo-stability and biocompatibility [126,127]. The schematic representation of multifunctional quantum dots is shown in Figure 9. 

The researchers created a novel formulation that includes graphene quantum dots conjugated with mesoporous silica nanoparticles (MSN) to provide a synergistic chemo-photothermal treatment. The GQD-MSN-DOX combination’s particle size was estimated to be between 50 and 60 nm. It also demonstrated temperature and pH-dependent drug release, as well as photothermal therapy generated by near-infrared irradiation, resulting in the formation of heat to destroy the malignant cells. This technology has also proven to be biocompatible and absorbed by 4T1 breast tumour cells. Chemo-photothermal therapy’s synergistic impact appears to be an excellent technique for cancer targeted therapy [128]. The applications of QDs are shown in Table 8.

## 4. Types of Targeting Moieties

Various targeting moieties are used for targeted delivery in cancer therapy, target moieties are commonly incorporated on surfaces of transporters by physical absorption or chemical reaction. Peptides, proteins, nucleic acids and small molecules (carbohydrates or vitamins) are examples of targeting moieties. 

### 4.1. Aptamer-Based Targeting 

Nucleic acid-containing ligands are known as aptamers that can bind to highly precise sites for drug molecule delivery. These aptamers can be identified by the ligand known as SELEX ligand. An example of aptamer-based targeting is the delivery of cisplatin to prostate cancer cells by using an aptamer conjugated on the surfaces of nanocarriers [130].

One of the most well-known aptamers for cancer treatment is AS1411 (single strand aptamer). It was shown to effectively limit the growth of a variety of human tumour cell lines, including prostate cancer, breast cancer and lung cancer. For effective cellular transport of AS1411, nanocarriers such as Apt-AuNS (aptamer conjugated gold nanoparticles) were used to increase the bioactivity of AS1411 [131]. 

### 4.2. Small Molecule-Based Targeting

Small compounds are inexpensive to create, used for targeting and have a limitless number of structures and properties. Folate is the most commonly investigated small molecules for drug delivery. Folate is an aqueous soluble vitamin B6 that is essential for men’s cell growth and division, particularly during embryonic development [2,132]. Riboflavin is a required nutrient for the cell metabolic process and a riboflavin carrier protein (RCP) has been found to be substantially increased in active tumour cells. An endogenous RCP ligand, flavin mononucleotide (FMN), was employed as a small molecule that targets the ligand in active tumour or endothelial cells [2].

Lactose-doxorubicin (Lac-DOX) based nanocarriers were developed and used for targeting cancer cells. The developed formulation exhibits improved anticancer activity and weak adverse effects by passive and active tumour targeting. Lac-DOX nanoparticles have extremely low toxicity in vivo, as seen by decreased uptake in normal body weights, key organs and normal blood biochemistry indices [133].

### 4.3. Peptide Based Targeting

They are ideal for targeting molecules due to their small low production cost, size and minimal immunogenicity. These peptides are derived from the binding areas of the protein of interest. A common example is ANGIO PEP-2, a peptide sequence and its complementary ligand is receptor-related protein (LRP), a type of low-density lipoprotein that is expressed in multiforme glioblastoma and blood–brain barrier (BBB), which is not an operable type of pituitary tumour. When coupled, the peptide sequence ANGIO PEP-2 will penetrate the BBB in sufficient quantities to target glioma in the brain [134,135].

Albumin fused chimeric polypeptide conjugated with self-assembled micelles were created by Parisa Y et al. and micelles are loaded with doxorubicin. When compared to conventional DOX, this formulation provides complete tumour inhibition with greater pharmacokinetics and dosage tolerance [136].

### 4.4. Antibody-Based Targeting

In recent decades, ligand manufacturing has been focused on the antibody’s classes. Within a single molecule that contains two binding epitopes and the target of interest has an unusually high level of affinity and selectivity. Rituximab is an antibody approved by FDA for non-lymphoma Hodgkin’s treatment [137]. Bevacizumab, is an anti-vascular endothelial growth factor (VEGF) monoclonal antibody used to treat metastatic rectal, breast and colon cancer, stops angiogenesis by sequestering soluble VEGF and inhibiting antibodies targeting different epitopes of the same protein from binding to VEGFR-2 [138]. 

Triple single chain antibodies were coupled to magnetic iron oxide nanoparticles to target pancreatic cancer for imaging and therapy were studied by Zou et al. Both in vitro and in vivo studies shows that triple single chain antibodies have clinical potential in both cancer therapy and imaging [139].

## 5. Stimulus for Drug Release 

The two types of stimuli are endogenous and exogenous. Exogenous stimulation is defined as an extra-corporal signal that causes medications to be released from smart-nanocarriers, such as a temperature change, an electric field, ultrasonic waves, or magnetic field. An endogenous stimulus is a signal created from within the body that causes the release of anti-cancer medications. Endogenous stimuli include pH changes, enzyme transformations, temperature changes and redox reactions [50].

### 5.1. Endogenous Stimulus

Intrinsic stimulus, also known as endogenous stimulation, is a type of stimulus that originates from the body. The triggering signal is generated by the body’s internal enzyme activity, pH level and redox activity in the case of endogenous stimulation. The following are detailed information on the many types of endogenous stimuli [140].

#### 5.1.1. pH-Responsive Stimulus DDS 

The Warburg effect states that tumour cells produce the majority of their energy in the cytosol via increased glycolysis followed by lactic acid fermentation [141]. This increased acid production causes cancer cells to have a lower PH. As pH levels differ from organ to organ and even tissue to tissue, the pH-responsive medicine delivery mechanism is unique. Tumours have an acidic pH compared to a slightly basic intracellular (pH 2). The inflammatory and extracellular tissues of tumours have a pH of about 6.5, while normal tissues have a pH of 7.4. The cytoplasm or organelles have lower pH, for-example lysosomes (pH 4–5), endosomes (pH 5–6) and the Golgi complex (pH 6.4). In conclusion, the pH differences between normal and cancer cells offers a solid foundation for creating a stimuli-sensitive drug delivery system [142,143]. The delivery system construction techniques fall into two categories based on the changes in the pH gradient outside and within the cells: One example is the polymer’s variations in conformation or dissolution behaviour under different pH environments [144,145,146,147]. The other possibility is that the delivery systems will dissolve due to the breakage of groups that are acid-stimuli in the nanocarriers, and as a result, targeted delivery at certain locations is possible [148,149,150,151].

Liu et al. have developed a mesoporous silica nanoparticle conjugated with chitosan. Chitosan is a smart drug delivery system, and this system releases the drug at narrow pH. Ibuprofen release was higher at pH 6.8 than pH 7.4 and pH-stimulus drug release of Ibuprofen for breast cancer has been accomplished [152].

#### 5.1.2. Redox-Sensitive Stimulus DDS 

Reductive compounds found in the human body include glutathione (GSH), vitamin E and vitamin C [153,154,155,156]. Based on the properties of these compounds, several redox-sensitive nanocarriers are produced and used in the controlled release of genes, proteins and anti-cancer medicines, targeted delivery and also for ultrasound imaging [157,158,159,160]. Zhao et al. (2015) used surface modification technology to create a redox responsive nanocapsule that could hold two functional molecules, one of which is encoded via disulphide bonds in the shell of the capsule and the other of which is enclosed in the capsule’s core. The redox reaction trigger could cause a cascade release of the loaded medication [161].

Sun et al. have developed an amphiphilic conjugate coupled heparosan with deoxycholic acid via disulfide bond self-assembled into stable micelles to deliver doxorubicin into cancer tissues. This formulation exhibited good loading capacity and glutathione-triggered drug release behaviour [162].

#### 5.1.3. Enzyme Responsive Stimulus DDS 

Phosphor esters, polymers and inorganic materials, among other nanomaterials, have previously been employed to develop enzyme responsive drug delivery systems [163,164,165,166,167]. In pathological conditions such as tumours or inflammations, the peptide structure or ester bonds of the stimuli-responsive carriers may be broken down by various enzymes, allowing the loaded medications or proteins to be released at specific sites to exhibit therapeutic effects [168,169]. The protein and peptides are degraded by an enzyme known as proteases, are an excellent choice for drug release from liposomes [3]. 

Lee et al. have prepared doxorubicin (Dox) loaded GLFG (Gly Leu-Phe-Gly) liposomes. These liposomes are degraded by cathepsin B enzyme, which is overexpressed in several cancer cells types and exhibits an effective anticancer effect on Hep G2 cells in vitro and inhibit cancer cell proliferation in a zebrafish model [170]. 

### 5.2. Exogenous Stimulus 

Ultrasound, temperature, magnetic field and light are the most common exogenous physical stimulus. Drug releases can happen quickly when these signals interact with nanocarriers that respond to external stimuli [171,172,173,174,175].

#### 5.2.1. Temperature Responsive Stimulus DDS 

Liposomes, nanoparticles and polymer micelles are common temperature-responsive carriers. When the ambient temperature exceeds the polymer’s critical solution temperature (CST), the hydrophilic–hydrophobic equilibrium breaks and the polymer chain dehydrates, causing the drug-delivering carrier’s structure to change and the contents packed in the system to be released [176].

Allam et al. have developed camptothecin loaded superparamagnetic nanoparticles (spions) coated with 1,2-Dipalmitoyl-sn-glycero-3-phosphocholine (DPPC) and l-α-dipalmitoylphosphatidyl glycerol (DPPG). This thermo-responsive nanocomposite has shown improved solubility and stability due to magnetic hyperthermia and also highly cytotoxicity towards cancer cells than the free camptothecin [177].

#### 5.2.2. Light-Responsive Delivery Systems

The precise drug release is achieved in light-responsive drug delivery systems when exposed to exogenous light (such as visible, infrared light or ultraviolet) [178,179,180,181,182]. 

For example, the doxorubicin-loaded gold nanocarrier has increased drug release under 808 nm illumination [183].

For chemophotothermal treatment in breast cancer, A. Zhang et al. have produced polyethylene glycol (PEG) linked liposomes (PEG-liposomes) coated doxorubicin-loaded mesoporous carbon nanocomponents. The study was carried in the presence and absence of NIR irradiation. The presence of NIR irradiation triggers the drug release from the formulation compared to the absence of NIR irradiation. The created system was able to transport the drug to breast cancer cells and cell toxicity viability tests revealed that the drug-loaded system had no cytotoxicity to normal cells [184].

#### 5.2.3. Magnetic Field Responsive DDS

An extracorporeal magnetic field is employed in magnetically induced systems to collect drug-loaded nanocarriers in tumour locations following nanocarrier injection. Magnetic stimulus candidates include core-shell shaped nanoparticles coated with magneto liposome (maghemite nanocrystals enclosed in liposomes), polymer or silica [185,186].

For siRNA delivery to breast cancer cells, superparamagnetic iron oxide nanoparticles coated with calcium phosphate and PEG-PAsp were developed by Dalmina et al. These systems efficiently carried siRNA and delivered the siRNA in breast cancer cells under an external magnetic field. This research vocation signifies VEGF (vascular endothelium growth factor) silencing being effective in breast cancer cells without causing cytotoxicity [187].

#### 5.2.4. Ultra-Sound Responsive DDS

Due to its non-ionizing irradiation, non-invasiveness and deep penetration into the body ultrasound are being studied extensively for medication release from nanocarriers [188]. Ultrasound can be used to create both mechanical and thermal effects in nanocarriers, allowing the loaded medicine to be released in 2007, Dromi et al. utilized high-intensity focused ultrasound waves to study temperature-sensitive liposomes for the drug release [189,190,191].

For hepatocellular carcinoma (HCC), Yin et al. have developed siRNA and paclitaxel (PTX) ultrasound sensitive nanobubbles (NBs). Encapsulating both anti-cancer drug paclitaxel (PTX) and siRNA into liposomes. When the low-frequency ultrasound was exposed, this system exhibits cell apoptosis decreases the tumour volume. As a result, new ways for co-administration of siRNA and PTX using ultrasound responsive polymer for hepatocellular carcinoma treatment have been created [192,193].

## 6. Nanomedicines: Development, Cost-Effectiveness and Commercialization

Though the nanotechnology and nanocarriers-based drug delivery approaches have gained much attention and popularity in today’s world and hold great potential from the application perspective, still there exists a lag between the development of excellent technology and its efficient commercialization. Presently, the commercialization of the majority of nanotherapeutics is either start-ups or small/medium-level enterprises driven. For emerging nanotherapeutics, there is a low interest of investment by big pharma firms. Hence, for the small nanomedical firms, it is an enormously difficult task to find a suitable major pharma firm for partnering; which will be willing to license and bring into the market their established nanotherapeutic technology [194]. Moreover, the firms dealing with nanomedicines are subject to suggestively higher per-unit costs. Subsequently, the prevailing diseconomies in the field for scale-up of nanomanufacturing ends in huge acquisition costs for nanotherapeutics; which ultimately hamper nanotherapeutics success and restricts their implication in day-to-day clinical practice [195]. Owing to low financial rewards allied with nanotherapeutic products, companies/firms developing and marketing such products find it difficult to recover their research and development costs. This signifies a major hurdle in the way of viable nanotherapeutics commercialization, thus undermining their future success in the market.

The unceasingly increasing healthcare costs are a prime challenge for both privately owned and governmental payers and development firms in the developed nations. At present, there is much pressure for delivering public services with utmost efficiency. Thus, medical developments in the future must not only be safe and efficacious, but should also have to be very cost-effective [196]. However, novel approaches that contain growing healthcare costs simultaneously maintaining clinical efficacy, seem to be almost inevitable [197]. However, the ‘expensive’ nanotherapeutics market uptake can be significantly increased by implying comprehensive standardized cost-effectiveness analyses [198]. Presently, such studies in the nanomedicine field are still in their infancy. The use of cost-effectiveness analyses and studies are indeed the vital missing link that could significantly improve the nanotherapeutics market introduction. Chiefly, it could be more crucial during the times when the healthcare sector is dealing with a shrinking budget [199]. On proper evaluation, the initially perceived ‘unattractive’ nanotherapeutic products, via their high acquisition costs, could turn into the ideal product for reimbursement.

Nanotherapeutics could offer affordable care, offsetting their high acquisition cost elsewhere. The major plus is the lack of adverse effects that strongly favour novel encapsulated nanotherapeutics; resulting not only in savings the medical procedures to be undertaken, but also reducing hospitalization days and personnel costs, and permitting continuity of work by the patients [199,200]. This is a very valuable boon for society. These cost savings will be pivotal for the development of overall cost-effective nanotherapeutic products [201,202]. Thus, the implication of standardized cost-effectiveness studies is one unique way of making the nanomedicine market more fascinating and likely attracting huge investments from big pharmaceutical firms. Lately, a comprehensive study on nanotherapeutics cost-effectiveness indicated that nanomedicines for ovarian cancer therapy are not only quite cost-effective, but also cost-saving for society [200]. Thus, to accomplish a smooth introduction of nanotherapeutics into the market, many of such cost-effectiveness studies focusing on a range of nanotherapeutics are needed to be undertaken; which in turn will support higher reimbursements and efficient commercialization.

## 7. Future Perspectives in Cancer Treatment 

Cancer nanomedicines have extensively advanced in recent years. As a result, nanoparticles with the potential of targeted drug delivery when combined with customizable triggering capabilities will have a considerable influence on cancer therapy [203]. Cancer is a diverse, heterogeneous, and mysterious disorder; hence, some of the cancer types and allied aetiology are yet unknown. Furthermore, the pathophysiology and physical characteristics of cancer differ from person to person. Thus, demanding for personalized and customizable anti-cancer therapy; which in itself is a great challenge [3]. Stimuli-sensitive nanostructures and DNA-based nanostructures have a wide range of applications in tumour treatment and diagnostics. The DNA nanostructures that are stimulus sensitive and hybrid in nature; offers excellent specificity and numerous functionalities in drug delivery [204]. Such DNA nanostructures and stimuli-sensitive nanocarriers have been extensively studied nowadays and hold great future in terms of their application in augmenting cancer treatment effectiveness with decreased instances of unwanted effects on normal cells.

Additionally, cancer immunotherapy has proven to be a viable option for achieving a variety of immunomodulatory activities and as an alternative to currently available conventional immunotherapies [205]. In turn, the development of cancer vaccines based on tailored polymeric nanoparticles—which activate a variety of anti-tumour immune responses—would be an adequate alternative to replace existing therapy modalities. Thus, the encouraging features of polymeric nanoparticles and tailored polymeric nanostructures for next-generation cancer immunotherapy modulations would be a viable approach in customised cancer treatment.

## 8. Conclusions

Nanocarriers, being a current scientific sensation, have an imperative part in biological applications, particularly in the delivery of anticancer drugs. Nanotechnology is a rapidly expanding and advancing field with the potential to scan, track, identify and transfer medications to specific tumour target cells. When compared to traditional cancer chemotherapy, nanocarriers have shown a considerable improvement in drug therapeutic efficacy with a few adverse reactions. Nanocarriers provide an extended therapeutic circulation lifetime, repeated therapeutic delivery and regulated and targeted drug release under-stimulation. However, in order to overcome the side effects of nanocarriers, surface modification techniques and nano-formulation finetuning must be used to continuously improve their characteristics. Smart nanocarriers must be stable, biodegradable, non-toxic and capable of releasing suitable amounts of drugs to target the tumour location for an extended period of time in order to provide the most effective and safest treatment. Considering this, the nanocarriers are neatly constructed to release the medication at the desired site before being completely degraded. Nanocarriers-mediated diagnostic and therapeutic approaches hold great promises for augmented cancer therapy and hence, with further advancements, these systems will be extensively adopted for facilitated cancer therapy. In this article, the significance of the various categories of smart nanocarriers and their promising potential for site-specific drug delivery applications has been outlined in great detail.

## Figures and Tables

**Table 1:** Liposomal formulation approved by FDA for cancer therapy.

Sr. No.	Product Name	Type	Drug	Uses/Treatment	Ref.
1	Vyxeos^®^	Liposome	Daunorubicin and Cytarabine	Acute myeloid leukaemia	[38]
2	Doxil^®^	PEGylated liposome	Doxorubicin	Ovarian and breast cancer	[39]
3	Lipo-Dox^®^	PEGylated liposome	Doxorubicin	Multiple myeloma, Ovarian and breast cancer	[40]
4	Onivyde^®^	PEGylated liposome	Irinotecan	Metastatic pancreatic cancer	[41]
5	Marqibo^®^	Liposome	Vincristine sulfate	Acute lymphoblastic leukemia	[41]

**Table 2:** Dendrimer for cancer treatment in clinical trials [53].

S. N.	Formulation	Type	Drug	Uses/Treatment
1	PAMAM ^#^ dendrimer	Dual-drug loaded dendrimer	Cisplatin and small interfering RNA ^#^	Solid tumours
2	PAMAM-PEG ^#^ dendrimer	PEGylated dendrimer	Doxorubicin	Breast, bladder, ovarian, lung and thyroid cancer
3	Folic acid-PAMAM dendrimer	PPI ^#^-dendrimer	Methotrexate	Epithelial cancer
4	PAMAM-PEG dendrimer	PEGylated dendrimer	5-Flouro uracil	Pancreatic cancer

^#^ PAMAM dendrimer—Poly(amidoamine) dendrimer, PAMAM-PEG dendrimer—Poly(amidoamine)-poly(ethylene glycol), PPI-dendrimer—Poly(propylene imine) dendrimers, RNA—Ribonucleic acid.

**Table 3:** Polymeric micelle for cancer therapy in clinical trial or uses. Reproduced with permission from reference [62].

Sr. No.	Product Name	Type	Drug	Status	Uses/Treatment
1	NK105	PEG-PAA ^#^ micelle	Paclitaxel	Phase 2 or 3	Breast cancer, Gastric cancer
2	NK911	PEG-PAA micelle	Doxorubicin	Phase 3	Solid malignancies
3	NC-6004	PEG-Polyglutamic acid	Cisplatin	Phase 3	Pancreatic cancer
4	Genexol-PM	PEG-PLA ^#^ micelle	Paclitaxel	FDA ^#^ Approved	Breast cancer, ovarian and lung cancer

^#^ PEG-PAA micelle—Poly(ethylene glycol)-polyacrylic acid, PEG-PLA micelle—Poly(ethylene glycol)-polylactide micelles, FDA—Food and Drug Administration.

**Table 4:** CNTs for cancer therapy. Reproduced with permission from reference [77].

S. N.	Type	Drug	Functionalization	Cancer Cells
1	SWCNTs ^#^	Doxorubicin & mitoxantrone	Polyethylene glycol, fluorescein, folic acid	HeLa cells
2	SWCNTs	7-Ethyl-10-hydroxycamptothecin (SN38)	Polyethylene glycol, antibody C225, folic acid	Colorectal cancer cells
3	SWCNTs	Doxorubicin	Folic acid, Chitosan & its derivatives (palmitoyl chitosan & carboxymethyl chitosan)	Human cervical cancer HeLa cells
4	MWCNTs ^#^	Doxorubicin	Polyethyleneimine, hyaluronic acid, fluorescein isothiocyanate	HeLa cells
5	MWCNT	Docetaxel, coumarin-6	d-Alpha-tocopheryl, polyethylene glycol 1000 succinate (TPGS), transferrin	Human lung cancer cells
6	MWCNTs	Doxorubicin	folic acid, Polyethylene glycol	HeLa cells

^#^ SWCNTs—Single walled carbon nanotubes, MWCNTs—Multi-walled carbon nanotubes.

**Table 5:** Applications of gold nanoparticles (AuNPs) in drug delivery for cancer therapy. Reproduced with permission from reference [88].

Types of Nanoparticles	Drug	Outcomes
Folate-AuNP ^#^	Cyclophosphamide	αHFR-positive ^#^ breast cancer cells were more sensitive to cyclophosphamide therapy.
MTX-AuNP ^#^	Methotrexate	Compared to free MTX, the MTX-AuNP have depicted higher cytotoxicity and tumour cell accumulation, as well as improved tumour inhibition.
VCR-AuNP ^#^	Vincristine (VCR)	Higher cytotoxicity and tumour cell accumulation compared to free VCR.
6MP-AuNP ^#^	6-mercaptopurine	Compared to 6MP alone, the 6MP-AuNP have greater antiproliferative effect.
5-FU-Glutathione-AuNP ^#^	5-Flourouracil	Compared to free 5-FU, the 5-FU-Glutathione-AuNP have greater anticancer effect.

^#^ Folate-AuNP—Folate-gold nanoparticles, MTX-AuNP—Methotrexate-gold nanoparticles, VCR-AuNP—Vincristine-gold nanoparticles, 6MP-AuNP—6-Mercaptopurine-gold nanoparticles, 5-FU-Glutathione-AuNP—5-Flourouracil-gold nanoparticles, αHFR—Alpha human folate receptor.

**Table 6:** Applications of MSNs using cancer models for improved cancer therapy. Reproduced with permission from reference [106].

Types of Nanoparticles	Drugs/Payloads	Applications/Outcomes
Magnetic MSNs ^#^- Neutrophils carrying	Doxorubicin	Precise diagnosis and high anti-glioma efficacy
MSNs- Poly-L-histidine and PEG coated	Sorafenib	Improved cancer therapy by PH trigger drug release
MSNs-CuS ^#^- Nanodots coated	Doxorubicin	Imaging and synergetic chemo-photothermal effect
MSNs-PEGylated lipid bilayer coating	Axitinib, celastrol	Improved cancer therapy
Organo MSNS- Polyethyleneimine coated	Doxorubicin P-gp SiRNA ^#^	Preventing multi drug resistance and promotion of chemotherapy

^#^ MSNs—Mesoporous silica nanoparticles, MSNs-CuS—Mesoporous silica nanoparticles-copper sulfide, P-gp—P-glycoprotein, SiRNA—Small interfering RNA.

**Table 7:** Superparamagnetic iron oxide nanoparticles (SPIONs) in use or under clinical trials for cancer therapy. Reproduced with permission from reference [116].

S. N.	Product Name	Formulation	Status	Application
1	Gastromark^®^	Aqueous suspension of silicone coated SPIONs	FDA-approved	Magnetic resonance imaging
2	Feridex^®^	SPIONs coated with dextran	FDA-approved	Magnetic resonance imaging
3	Feraheme^®^	SPIONs coated with polyglucose sorbitol carboxymethylether	FDA-approved	Magnetic resonance imaging
4	NCT01270139	Iron bearing nanoparticles	Clinical trial	Hyperthermia
5	NCT01436123	Gold nanoparticles with iron oxide-silica shells	Clinical trial	Hyperthermia

**Table 8:** Applications of QDs in drug delivery for augmented cancer therapy. Reproduced with permission from reference [129].

S. N.	Delivery System	Purpose
1	Zinc oxide QDs	Liver cancer
2	GQD-mesoporous silica nanoparticle-DOX ^#^	PH dependent release + Photothermal therapy
3	Silicon dioxide -GQD-DOX ^#^	Cancer theragnostic
4	Nitrogen functionalized GQD-methotrexate	Breast cancer
5	GQD-Biotin-Doxorubicin	Targeting overexpressed biotin receptor for cancer therapy
6	Black phosphorous QDs-PEG ^#^	Combination of PTT ^#^ and PDT ^#^

^#^ GQD—Graphene quantum dots, GQD-DOX—Graphene quantum dots-Doxorubicin, QDs-PEG—Quantum dots-polyethylene glycol, PTT—Photothermal therapy, PDT—Photodynamic therapy.

## Data Availability

This study did not report any data.
